# Pathway analysis for family data using nested random-effects models

**DOI:** 10.1186/1753-6561-5-S9-S22

**Published:** 2011-11-29

**Authors:** Jeanine J Houwing-Duistermaat, Hae-Won Uh, Roula Tsonaka

**Affiliations:** 1 Department of Medical Statistics and Bioinformatics, Leiden University Medical Center, PO Box 9600, 2300 RC, Leiden, The Netherlands

## Abstract

Recently we proposed a novel two-step approach to test for pathway effects in disease progression. The goal of this approach is to study the joint effect of multiple single-nucleotide polymorphisms that belong to certain genes. By using random effects, our approach acknowledges the correlations within and between genes when testing for pathway effects. Gene-gene and gene-environment interactions can be included in the model. The method can be implemented with standard software, and the distribution of the test statistics under the null hypothesis can be approximated by using standard chi-square distributions. Hence no extensive permutations are needed for computations of the *p*-value. In this paper we adapt and apply the method to family data, and we study its performance for sequence data from Genetic Analysis Workshop 17. For the set of unrelated subjects, the performance of the new test was disappointing. We found a power of 6% for the binary outcome and of 18% for the quantitative trait Q1. For family data the new approach appears to perform well, especially for the quantitative outcome. We found a power of 39% for the binary outcome and a power of 89% for the quantitative trait Q1.

## Background

Testing for the joint effect of single-nucleotide polymorphisms (SNPs) located in a set of genes is a popular alternative to single-marker tests [1]. Typically these SNPs have small effect sizes, and thus separate SNP analysis methods will be underpowered. On the other hand, approaches that consider sets of genes and test for the combined effect of multiple SNPs will be more powerful. Gene sets can be defined on the basis of the biological function of the genes (pathways) and can contribute thereby to biologically interpretable results. Gene-set methods were originally proposed for gene expression data and have recently been adapted to test for pathway effects using genetic data [2,3]. A fundamental difference between gene expression data and genetic data is that in genetic data multiple SNPs within genes that are correlated are available. Current pathway-based methods for genetic data do not properly capture this correlation structure of the genetic data and therefore may lose efficiency. Recently, two pathway approaches were proposed that take the correlation between SNPs into account [4,5]. Both approaches have two steps: (1) reducing the dimensionality of the genetic data and producing gene-specific summaries and (2) introducing these summaries as covariates into the model for the phenotype.

The two-stage approach of Tsonaka et al. [5] models the correlation between SNPs in a pathway using a generalized linear mixed model for the SNPs with nested random effects. This approach uses a pathway-level and a gene-level random effect to capture the correlation between genes and within each gene, respectively. The empirical Bayes estimates of the random effects per subject and gene are used as summary measures of the SNP data and are included in the phenotype model to test for pathway association. Tsonaka and colleagues proposed this approach to test for pathway effects for disease progression in a longitudinal study. They used a likelihood ratio test and a Wald statistic and showed by simulations that the test statistics follow a chi-square distribution under the null hypothesis. The aim of this paper is to study the performance of this approach for the sequence data on the families of Genetic Analysis Workshop 17 (GAW17).

We knew the answers and took the simulation setup for these data into account. We considered that the genes that were simulated were associated with the phenotype Q1 and studied their association with the quantitative traits Q1 and Q4 and the binary trait. Because an interaction between smoking status and the *KDR* gene was included in the simulation model, we also considered this gene-environment interaction in the phenotype models.

## Methods

### Study sample

We considered data from 697 unrelated individuals and 311 subjects from 140 sibships forming 8 families. The sibship sizes vary from one to six siblings. The degree of relationships between members of different sibships was larger than 0.25; that is, parents were removed when their offspring were included. For the pathway analysis we considered SNPs that belong to the following eight genes: *ARNT*, *ELAVL4*, *FLT1*, *HIF3A*, *KDR*, *FLT4*, and *VEGFA*. Gene *HIF1A* was not considered because it does not contain SNPs that vary in the family data. In total, the analysis was restricted to 48 out of 125 SNPs in the Q1 (vascular endothelial growth factor [VEGF]) pathway because only these SNPs show variation in the families. The minor allele frequencies (MAFs) of the associated variants in these genes vary from 0.000717 to 0.164933. As covariates we considered Age and Smoking status. For gene *KDR*, an interaction with Smoking was included in the phenotype models. We applied the two-stage method to the 200 simulated GAW17 data sets [6] to study its power. In addition, we present the results of the analysis of data set 1. A description of data set 1 is given in Table 1.

**Table 1:** Description of data set 1

Trait	Unrelated subjects (*n* = 697)	Family (*n* = 311)
Q4 (mean and SD)	0 (1)	0.75 (0.58)
Q1 (mean and SD)	0 (1)	−0.25 (0.99)
Binary outcome (affected) (%)	30.0	11.6
Smoking (yes) (%)	26.0	21.9

### Model specification

Let *y_ij_* be the outcome variable for individual *j* from sibship *i*. Assume that a pathway is analyzed with *G* genes and that each gene *g* (*g* = 1, *…*, *G*) contains *S_g_* SNPs. Let *w_ijgs_* be the genotype at SNP *s* (*s* = 1, *…*, *S_g_*) located in gene *g* (*g* = 1, *…*, *G*) for individual *j* of sibship *i*. The genotype *w_ijgs_* is coded 0, 1, or 2. For individual *j* of sibship *i*, let  and  be vectors with covariate values for the phenotypes and genotypes. Within each gene SNPs are correlated, and in practice only a part of the SNPs will be associated with the outcome.

### Gene model

We assume that Hardy-Weinberg equilibrium holds. We consider three random levels: (1) a sibship’s random effect, (2) a subject’s random effect, and (3) a gene effect within a subject. Let *b_i_* be the random effect for the sibship, *b_ij_* the random effect for subject *j* within sibship *i*, and *b_ijg_* the random effect of gene *g* of person *j* within sibship *i*, and let  be a set of covariates. Note that the random effect *b_ij_* represents for each individual *j* the shared effect of the genes of the pathway. Given these random effects *b_i_*, *b_ij_*, and *b_ijg_* and the covariates , *w_ijgs_* is assumed to follow a binomial distribution with *n* = 2 trails and probability *π_ijgs_*. The probability *π_igs_* is modeled as follows:(1)

where *b_i_*, *b_ij_*, and *b_ijg_* follow normal distributions with zero mean and variances , , and , respectively. For unrelated subjects we use model (1) without the sibship effect *b_i_*.

For individuals and for each gene the empirical Bayes estimate is given by:(2)

Intuitively the value of the empirical Bayes estimate will increase with the number of rare variants that a subject carries.

### Phenotype model

The empirical Bayes estimates obtained from the first stage can be plugged into the models for the phenotypes to test for pathway effects and gene-specific effects. For the quantitative traits (i.e., Q1 and Q4) we use a linear mixed model:(3)

where *u_i_* is a normally distributed random sibship effect and *e_ij_* is a normally distributed residual. For the binary outcome variable a generalized estimating equation (GEE) approach was used with an exchangeable correlation structure for subjects within sibships:(4)

where *h* is the logit function. The lme4 package in R was used to fit mixed models. The gee package in R [7] was used for the GEE approach. Based on models (3) and (4) we can test the null hypothesis of no pathway effect, which is equivalent to testing the null hypothesis *H*_0_: *γ*_1_ = … = *γ_G_* = 0. We used a Wald statistic with *G* degrees of freedom. In addition, gene-level effects can be tested.

### Type I error

Using extensive simulations, Tsonaka et al. [5] showed that the test statistics preserve the type I error at a nominal level for pathway analysis for longitudinal data. We tested for association between the Q1 pathway and the Q4 trait. Because Q4 should not be influenced by the genes of this pathway, the power should be equivalent to the type I error.

## Results

### Type I error and power

We fitted model (1) to the 48 SNPs of the Q1 pathway, which showed variation in the families. We included the covariate Smoking in the model because an interaction between *KDR* and smoking status was included in the simulations. Then we plugged the empirical Bayes estimates per gene and subject into models (3) and (4) for the quantitative and binary variables, respectively. Age and Smoking were included as covariates. In addition, we included an interaction between smoking status and the empirical Bayes estimate for *KDR*.

In Table 2 for Q1, Q4, and the binary outcome, we show the percentages of the data sets for which the null hypothesis of no Q1 pathway is rejected at the 5% level. The results are based on all 200 data sets. The genes of the Q1 pathway have a direct effect on Q1 and, through Q1, also have an effect on the binary outcome. These genes should not be associated with Q4; hence the percentages for Q4 are estimates of the type I error. For unrelated subjects the type I error is near 5%, but for the family data the type I error is too high (10%). The novel test performs well for quantitative traits observed in families: The power to detect the Q1 pathway for Q1 is 89%. The power to detect the Q1 pathway for disease in families is smaller, namely, 39%. The power to detect the Q1 pathway in the set of unrelated subjects is small.

**Table 2:** Percentage of data sets for which *H*_0_: “no Q1 pathway effect” is rejected at the 5% level

Trait	Unrelated subjects (%)	Family (%)
Q4	5	10
Q1	18	89
Binary outcome	6	39

### Analysis of data set 1

The results of the pathway analyses for data set 1 are given in Table 3. In the families, we obtained a highly significant result for the pathway of eight genes for the quantitative trait Q1 (*p* = 4.9 × 10^−10^). In the set of unrelated individuals the pathway was not significantly associated with Q1 (*p* = 0.06). Also the *p*-values per gene are presented in Table 3. These *p*-values correspond to tests for a gene effect conditional on the empirical Bayes estimate of the remaining genes in the pathway. For the family data and Q1 trait, the *FLT4* and *VEGFC* genes were significant.

**Table 3:** *p*-Values for testing pathway and gene effects for data sets

Gene	Binary outcome		Quantitative trait Q1	
	
	Unrel^a^	FAM^b^	Unrel^a^	FAM^b^
*ARNT*	0.13	0.12	0.27	0.76
*ELAVL4*	0.14	0.55	0.31	0.15
*FLT1*	0.32	0.44	0.01	0.84
*FLT4*	0.77	0.11	0.32	1.6 × 10^−8^
*HIF3A*	0.69	0.62	0.07	0.04
*KDR*^c^	0.26	0.53	0.35	0.89
Interaction^d^	0.86	0.38	0.24	0.68
*VEGFA*	0.45	0.10	0.26	0.004
*VEGFC*	0.98	0.42	0.69	6.8 × 10^−7^
Pathway	0.51	0.002	0.06	4.9 × 10^−10^

^a^ Based on genotypes of 697 unrelated individuals.

^b^ Based on 140 sibships.

^c^ Two degrees of freedom tests (including interaction term).

^d^* p*-value for interaction term.

For the binary outcome in the families we found a significant association, although it was less strong than that for the quantitative trait (*p* = 0.002). None of the genes were significant, which suggests that multiple SNPs in multiple genes have a joint effect on the outcome. The Q1 pathway was not significantly associated with the binary trait in the unrelated individuals.

The interaction between smoking status and the *KDR* gene was not significant for both outcomes either in the families or in the set of unrelated subjects (see Table 3).

## Discussion

The pathway analysis applied to the family data resulted in more significant results than using the set of unrelated individuals, especially for the analysis of trait Q1. One reason for the better performance in the family data compared to the set of unrelated subjects is probably the oversampling of rare variants in the families. For example, the SNP C4S4935 of the *VEGFC* gene has a MAF of 0.0290 in the families in contrast to a MAF of 0.0007 in the set of unrelated individuals. Also, two of the three SNPs of the *FLT4* gene have a larger MAF in the families than in the unrelated set. Another reason for the larger power in the families is the fact that we are testing against a smaller residual when a sibship effect is included. Finally, the power may be too high because the size of the test is not correct. Indeed, based on the pathway analysis for trait Q4, we obtained a high type I error.

We further investigated whether the high type I error could be attributed to the fact that correlation between cousin pairs is not taken into account in our models (3) and (4). Thus we added an extra random effect to these models, but the obtained type I error did not change. Uh et al. [8] also obtained too large type I errors when testing associations between Q1 genes and the Q4 trait in the unrelated subjects. They showed that, by using simulations under the null hypothesis, the type I error of the test statistics are at a nominal level. One reason for the large percentage of rejections using Q4 may be that Q1 and Q4 are not independent in the data sets. Indeed in all 200 data sets Pearson’s correlation coefficients between the two traits are smaller than 0 (mean, −0.31; minimum, −0.39; and maximum, −0.24). Concerning the binary trait, the power to detect the Q1 pathway was smaller than for Q1 trait. The reasons are that the Q1 genes have an indirect effect on the binary trait and that we used a less efficient approach, namely, the GEE approach instead of a likelihood-based approach.

The power to detect the pathway in unrelated subjects was disappointing. The rare variants have smaller frequencies in these samples and are not tagged by more common variants [8]. The power of a set of unrelated individuals may be improved by combining estimates from different studies, for example, the GAW17 unrelated individuals and family data [9]. For GAW17 this approach could not be applied because the rare variants are oversampled in the families. Another approach may be to focus on rare variants only. One of the reviewers pointed out that an alternative test for association between the outcome and rare variants can be obtained by fitting model (1) to all rare variants of all genes of the pathway. By doing so, one can obtain an empirical Bayes estimate that represents all rare variants of the pathway. The advantage of this approach is that by including this empirical Bayes estimate in the phenotype model, a one degree of freedom test for association between the rare variants and phenotypes is obtained that may be more powerful than the *G* degrees of freedom test that was studied in this paper. A disadvantage of this approach is that the structure of SNPs within genes is ignored. Therefore gene-environment interaction between specific genes and environmental factors cannot be modeled. For the GAW17 data, interaction between *KDR* and smoking status was included in the simulation model.

Our novel method captures the correlation between SNPs within and between genes by using random effects. Another approach was proposed by Chen et al. [4]. They summarize the SNPs per gene by using principal components (eigen-SNPs). They show that their approach is more powerful than methods that ignore the dependency structure between the SNPs. This approach cannot be directly applied to family data because one of the principal components may capture the dependence between relatives. Therefore the selection of eigen-SNPs to represent a gene is not straightforward. Moreover, SNPs are categorical variables, and therefore applying principal components analysis may not be optimal. Finally, principal components analysis cannot deal with missing genotypes. Hence missing genotypes should be first imputed before this approach can be applied.

Application of the new method to sequence data in unrelated individuals shows that when rare variants are not tagged by common variants, the new method is not able to detect these rare variants. Currently we are working on a method that jointly models the rare variants by using collapsing methods and the common variants.

## Conclusions

Our novel pathway test is a powerful tool to detect pathways in family data. The advantages of this method are that it captures the correlation between SNPs, can deal with missing data, can adjust for gene-gene or gene-environment interaction, can be applied to any phenotype model, and can be implemented in standard statistical software.

## Competing interests

The authors declare that there are no competing interests.

## Authors’ contributions

JJHD: method development and writing of manuscript; HWU: data management and contributed to discussion on methodology and results of data analyses; and RT: statistical analyses, method development and helped to draft the manuscript
